# ARHGEF7 (BETA-PIX) Acts as Guanine Nucleotide Exchange Factor for Leucine-Rich Repeat Kinase 2

**DOI:** 10.1371/journal.pone.0013762

**Published:** 2010-10-29

**Authors:** Karina Haebig, Christian Johannes Gloeckner, Marta Garcia Miralles, Frank Gillardon, Claudia Schulte, Olaf Riess, Marius Ueffing, Saskia Biskup, Michael Bonin

**Affiliations:** 1 Institute of Human Genetics, Department of Medical Genetics, University of Tuebingen, Tuebingen, Germany; 2 Department of Protein Science, Helmholtz-Zentrum München, Munich, Germany; 3 Center for Ophthalmology, Institute for Ophthalmic Research, University of Tuebingen, Tuebingen, Germany; 4 Hertie Institute for Clinical Brain Research and German Center for Neurodegenerative Diseases, University of Tuebingen, Tuebingen, Germany; 5 Boehringer-Ingelheim Pharma GmbH & Co. KG, CNS Research, Biberach an der Riss, Germany; Brigham and Women's Hospital, Harvard Medical School, United States of America

## Abstract

**Background:**

Mutations within the leucine-rich repeat kinase 2 (LRRK2) gene are a common cause of familial and sporadic Parkinson's disease. The multidomain protein LRRK2 exhibits overall low GTPase and kinase activity *in vitro*.

**Methodology/Principal Findings:**

Here, we show that the rho guanine nucleotide exchange factor ARHGEF7 and the small GTPase CDC42 are interacting with LRRK2 *in vitro* and *in vivo*. GTPase activity of full-length LRRK2 increases in the presence of recombinant ARHGEF7. Interestingly, LRRK2 phosphorylates ARHGEF7 *in vitro* at previously unknown phosphorylation sites. We provide evidence that ARHGEF7 might act as a guanine nucleotide exchange factor for LRRK2 and that R1441C mutant LRRK2 with reduced GTP hydrolysis activity also shows reduced binding to ARHGEF7.

**Conclusions/Significance:**

Downstream effects of phosphorylation of ARHGEF7 through LRRK2 could be (i) a feedback control mechanism for LRRK2 activity as well as (ii) an impact of LRRK2 on actin cytoskeleton regulation. A newly identified familial mutation N1437S, localized within the GTPase domain of LRRK2, further underlines the importance of the GTPase domain of LRRK2 in Parkinson's disease pathogenesis.

## Introduction

The multidomain protein kinase LRRK2 is an attractive therapeutic target as it was shown that this previously unknown kinase can cause Parkinson's disease (PD) if mutated [1], [2]. Mutations within LRRK2 contribute to 5-6% of all cases of autosomal-dominant as well as to 1-2% of cases of sporadic Parkinson's disease. Pathogenic mutations are primarily found in the enzymatic domains of LRRK2, the GTPase and the kinase domain. The pathogenic mechanism and the normal function of the protein LRRK2 are far from being understood [3], [4], [5], [6]. West et al. were the first to show that mutated LRRK2 exhibits increased kinase activity *in vitro* [7]. This important finding led to numerous investigations on how LRRK2 kinase activity might be regulated. Current evidence suggests dimerization [8], [9], [10], [11], autophosphorylation [7], [8], [9], [12], [13], [14], [15], [16], [17], [18], [19] and intramolecular signaling through the GTPase domain of LRRK2 itself [10], [13], [20], [21], [22], [23]. Since activity of LRRK2 *in vitro* is rather small compared to known GTPases and kinases the question remains if normal LRRK2 primarily acts through its enzymatic activities or rather serves as a scaffold protein regulating cellular processes by orchestrating protein complexes. The discovery of endogenous interactors of LRRK2 was greatly limited through the lack of specific antibodies. We previously used siRNA knockdown in SH-SY5Y to gain further insight into pathways regulated by LRRK2 [24]. Interestingly, we found a deregulation of actin-cytoskeleton signaling cascades with the guanine nucleotide exchange factor ARHGEF7 and the small GTPase CDC42 being the most significantly up-regulated genes. Several lines of evidence suggest an involvement of LRRK2 in regulation of neurite outgrowth [25], [26]. We therefore characterized these central regulators of the cytoskeleton as potential interactors of LRRK2.

## Results

### ARHGEF7 and CDC42 interact and partially co-localize with LRRK2

**Figure 1:**
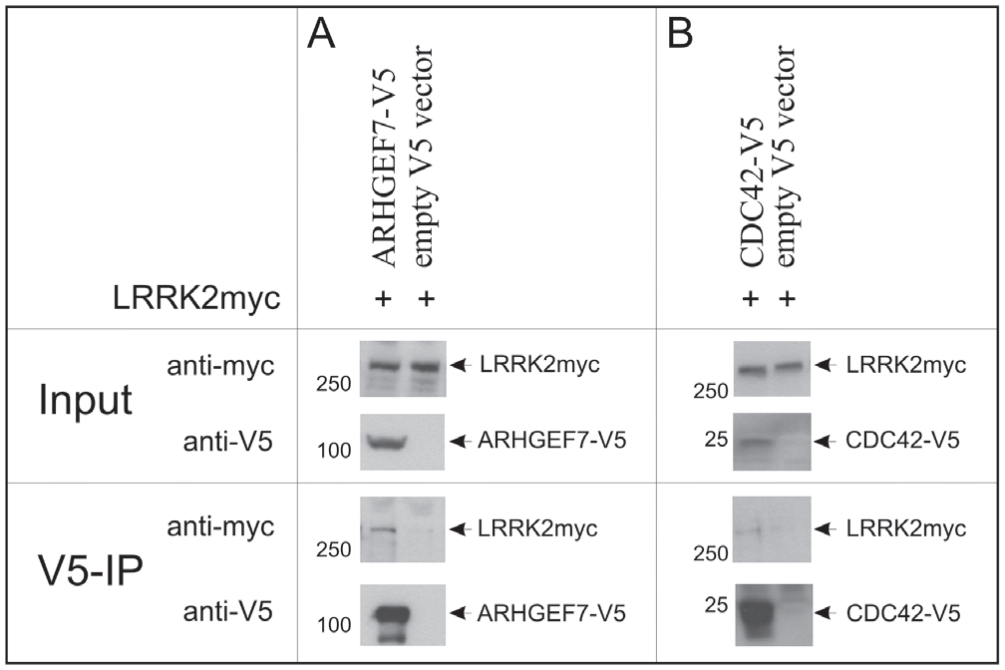
Co-immunoprecipitation of full-length LRRK2 with ARHGEF7 (A) and CDC42 (B). Myc-tagged full-length LRRK2 and the indicated V5-tagged constructs (A: ARHGEF7, B: CDC42) were co-transfected in HEK293 cells and analyzed in V5-Co-immunoprecipitation (V5-IP). 60 µg of protein lysate were used as input control. Immunoblotting was performed using V5 and myc antibodies. Empty V5 vector was used as negative control for each condition.

First, we generated V5-tagged ARHGEF7 and CDC42 and tested these proteins for interaction with co-expressed full-length myc-tagged LRRK2 in HEK293 cells. A robust interaction of LRRK2 with both proteins was demonstrated (Figure 1). Over-expressed LRRK2 also interacts with ARHGEF7 and CDC42 in SH-5YSY cells (data not shown).

**Figure 2:**
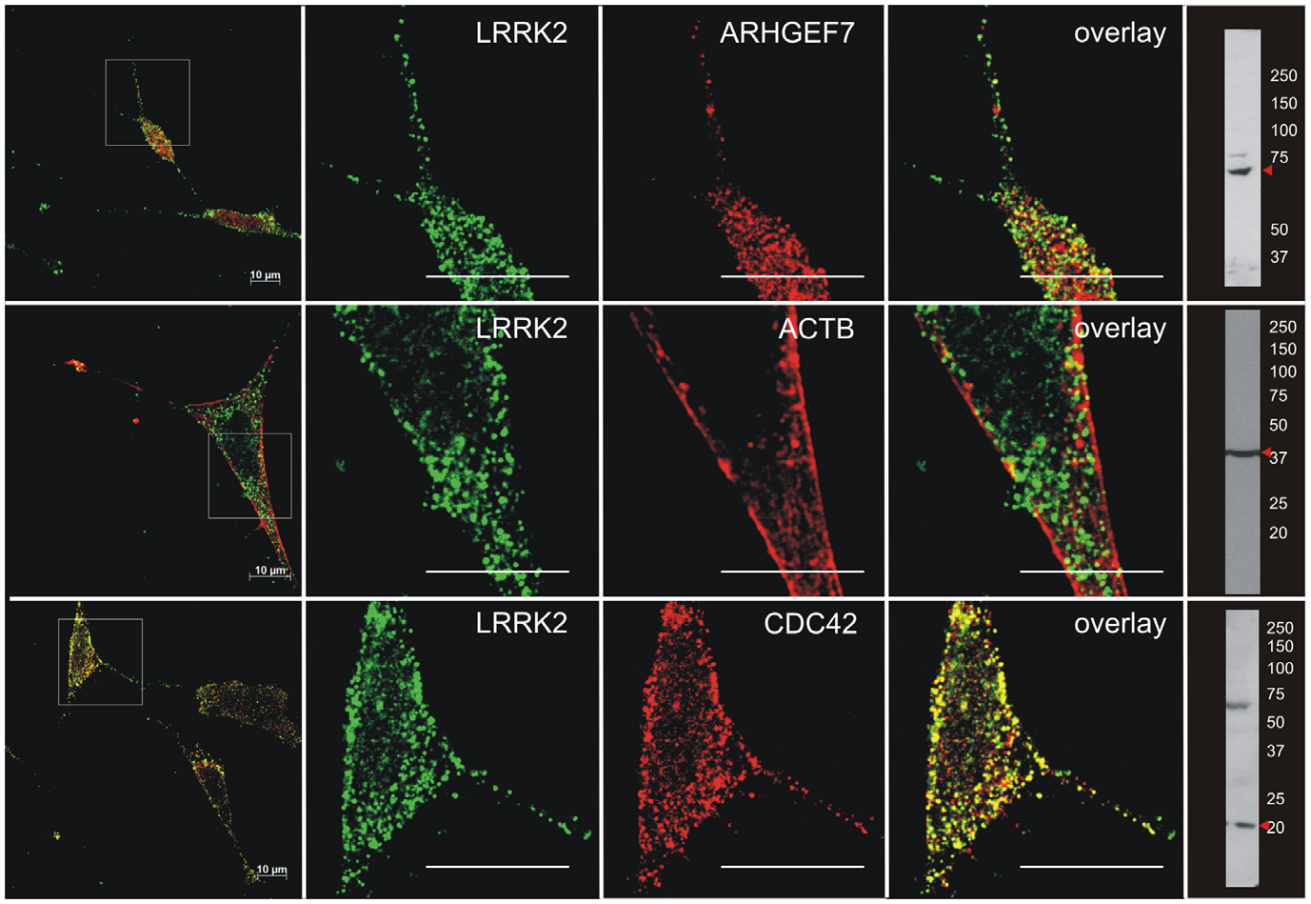
Co-localisation of LRRK2 with ARHGEF7, CDC42 and ACTB in differentiated SH-SY5Y cells. Differentiated SH-SY5Y cells were double stained for endogenous LRRK2-CY2 and ARHGEF7-CY3, ACTB-CY3 or CDC42-CY3 and analyzed with confocal microscopy (LSM510, Zeiss). Specificity of the antibodies is indicated by immunoblotting. The specificity of the Novus 267 LRRK2 antibody is shown by means of RNAi (Figure S1). Scale bar  = 10 µm.

Next, we investigated if endogenous LRRK2 (detected with the Novus 267 LRRK2 antibody) co-localizes with endogenous ARHGEF7, CDC42 and ACTB in SH-SY5Y. Partial co-localisation of all three proteins is shown in retinoic acid differentiated SH-SY5Y cell bodies and neurites supporting a potential *in vivo* relevance of these interactions, respectively (Figure 2). Specificity of the antibodies was demonstrated by immunoblotting (Figure 2 and Figure S1).

**Figure 3:**
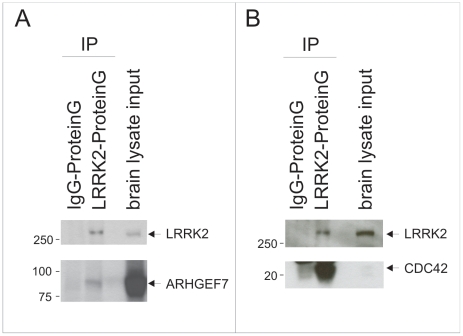
Interaction analysis of ARHGEF7, CDC42 and LRRK2 from mouse brain lysates. Lysate of whole mouse brain was used for pulldown of endogenous LRRK2 on ProteinG agarose beads. Immunoblotting with specific antibodies for endogenous proteins indicates the enrichment of LRRK2 on the beads as well as the coupling of the interacting proteins ARHGEF7 (A) and CDC42 (B). Coupling of IgG on ProteinG agarose beads and incubating with the same lysate was used as negative control.

The endogenous interaction of LRRK2 with ARHGEF7 and CDC42 in mouse brain lysates could robustly be demonstrated by co-immunoprecipitation using a monoclonal anti-LRRK2 antibody described by Bauer and colleagues ([27], Figure 3). Figure S2A shows that the guanine nucleotide exchange factor SOS1 did not interact with LRRK2. As positive control we confirm the already known interaction between LRRK2 and γ-tubulin (TUBA, [28], [29], [30]), here also shown in mouse brain lysate (Figure S2B).

### Reduced interaction between ARHGEF7 and R1441C LRRK2 mutant in HEK293 cells

**Figure 4:**
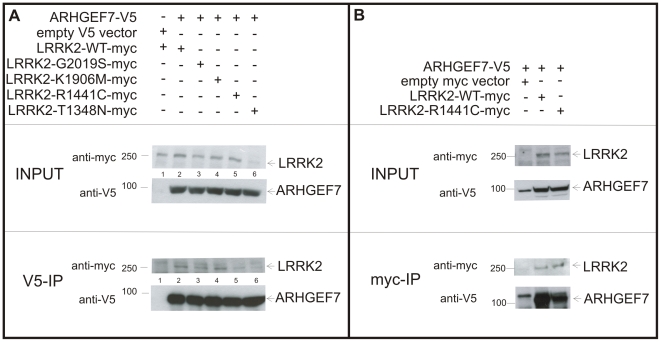
Co-immunoprecipitation of ARHGEF7 with mutated LRRK2. (A) Myc-tagged full-length LRRK2 with different mutations (WT, G2019S, K1906M, R1441C, T1348N) was co-transfected with V5-tagged ARHGEF7 in HEK293 cells and subjected to V5-Co-immunoprecipitation. Transfection with empty V5 vector was used as negative control (lane 1). 60 µg of protein lysates were used as input control. Immunoblotting was performed using antibodies against V5 and myc. Interaction strength between ARHGEF7 and mutated LRRK2 (lane 3–6) was compared to WT LRRK2 (lane 2). (B) Confirmation of reduced binding between ARHGEF7 and full-length R1441C LRRK2 in comparison to full-length WT LRRK2 using myc-IP.

Next, we analyzed if the known pathogenic mutations R1441C and G2019S affect the interaction between ARHGEF7 and LRRK2. As controls a GTPase-impaired version (T1348N) and a kinase-dead version (K1906M) of LRRK2 were used, respectively. As shown in Figure 4A (lane 2, 3 and 4) there is no difference between WT and G2019S and kinase-dead LRRK2 interacting with ARHGEF7 pulldowned with V5-agarose beads. Clearly, the R1441C mutation in LRRK2 leads to a reduced binding between R1441C mutant LRRK2 and ARHGEF7 (lane 5 compared to lane 2). This result was confirmed with an inverse co-immunoprecipitation using myc-agarose beads coupling LRRK2 and pulling down ARHGEF7 (Figure 4B). Although expression levels of the potentially instable T1348N mutant (Figure 4A, lane 6) are less compared to other mutants, interaction still takes place. The quantification is shown in Figure S3.

### ARHGEF7 enhances GTP binding of R1441C-LRRK2

**Figure 5:**
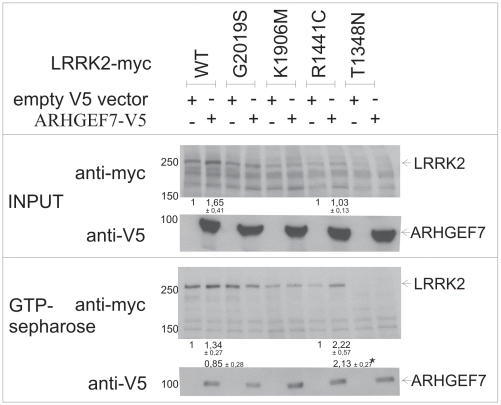
LRRK2-GTP-binding influence through ARHGEF7. GTP-binding efficiency of full-length myc-tagged LRRK2 (WT, G2019S, K1906M, R1441C, T1348N) on GTP-sepharose was analysed in comparison between co-expression of empty V5 vector or V5-tagged ARHGEF7. Immunoblotting was performed with V5 and myc antibodies. Differences were analysed by pixel densitometry and were indicated. Errors are SEM. *p-value ≤0.05.

It has been previously shown that LRRK2 without guanine nucleotide exchange factors (GEFs) or GTPase-activating proteins (GAPs) preferentially binds to GTP versus GDP [12], [23], [31]. We therefore examined if ARHGEF7 can influence the affinity of LRRK2 to GTP-sepharose. Co-expression of ARHGEF7 with WT LRRK2 leads to an induction of LRRK2 protein expression (Figure 5, lane 2 compared to lane 1); a finding that is consistent with increased GTP binding of LRRK2. We then tested the G2019S (Figure 5, lane 3 and 4) and K1906M (Figure 5, lane 5 and 6) mutation and did not detect any influence of LRRK2 GTP binding when co-expressing ARHGEF7. As shown by others the T1348N mutation albeit with low expression levels, is not able to bind GTP (Figure 5, lane 9/10). Interestingly, the R1441C mutation if co-expressed with ARHGEF7 leads to a significant two-fold increase in GTP binding affinity to LRRK2 (Figure 5, lane 8 compared to lane 7). The quantification is shown in Figure S4.

### ARHGEF7 acts as a guanine nucleotide exchange factor of LRRK2 in HEK293 cells

**Figure 6:**
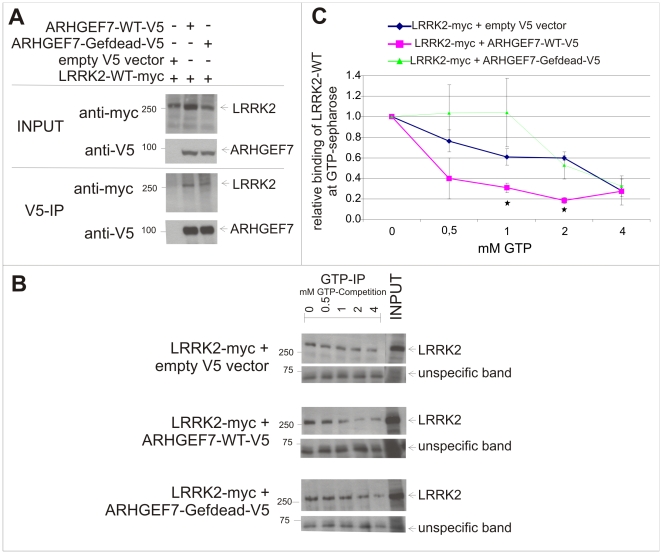
Interaction of GEF-dead ARHGEF7 with LRRK2 (A) and Influence of ARHGEF7 on GTP exchange capacity of LRRK2 (B). (A/B) Co-expression of full-length myc-tagged LRRK2 and either ARHGEF7-WT-V5 or ARHGEF7-GEF-dead-V5 or empty V5 vector as negative control in HEK293 cells and subsequent analysis of interaction strength with V5-Co-immunoprecipitation. 60 µg of protein lysates were used as input control. Immunoblotting was performed using myc or V5 antibodies. (B/C) Same transfections as in (A) were used for GTP exchange analysis on GTP-sepharose (GTP-IP) with competition of 0 (as baseline), 0.5, 1, 2, 4 mM soluble GTP. Detection with myc antibody is used to analyse the bound full-length LRRK2 on GTP-sepharose beads (B). Quantification of pixel densities was performed of three independent experiments. A nonspecific band was used as loading control. (C). Error bars  =  SEM, *p-value ≤0.05.

To further analyse the functional impact of the ARHGEF7 - LRRK2 interaction we tested if a GEF-dead variant of ARHGEF7 [32] is still able to bind LRRK2. In Figure 6A we show that the guanidine nucleotide exchange activity of ARHGEF7 is not relevant for binding to LRRK2 in general. Other groups previously demonstrated that 2 mM GTP is able to reduce the binding of LRRK2 to GTP-sepharose beads [12], [31]. We performed a competition assay using four different concentrations of soluble GTP with 0 mM GTP as baseline. We estimated the strength of LRRK2 binding to GTP mediated by ARHGEF7 indicated by fewer amounts of GTP to compete with GTP-sepharose for the binding to LRRK2. As expected co-expression of full-length myc-tagged LRRK2 and empty V5 vector leads to reduced binding of LRRK2 to GTP-sepharose with increasing amounts of soluble GTP (Figure 6B). Co-expression of full-length myc-tagged LRRK2 with wild-type ARHGEF7 leads to reduced LRRK2 binding on GTP-sepharose beads at already much lower concentrations of soluble GTP. Compared to conditions without expressing ARHGEF7 there is a significant difference in bound LRRK2 on GTP-sepharose beads at 1 mM and 2 mM soluble GTP respectively (Figure 6C). Co-expression of full-length myc-tagged LRRK2 with a GEF-dead variant of ARHGEF7 does not enhance GTP exchange of GTP-bound LRRK2. With this qualitative analysis we conclude that ARHGEF7 has the potential to act as a guanine nucleotide exchange factor for LRRK2.

### GTPase activity of full-length LRRK2 is increased in the presence of recombinant ARHGEF7 *in vitro*

**Figure 7:**
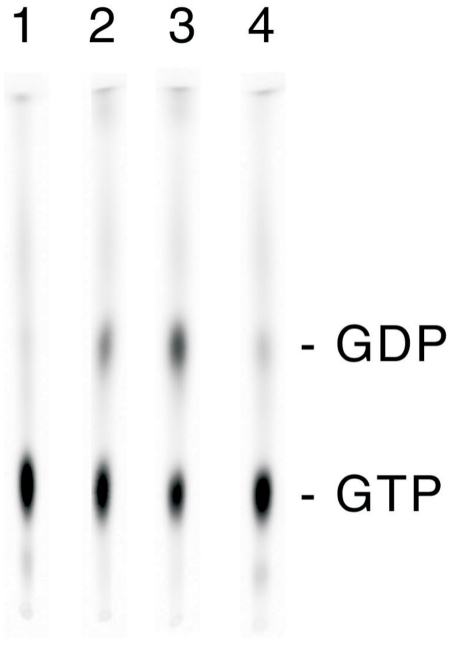
ARHGEF7 augments GTPase activity of full-length LRRK2. Hydrolysis of [α-^33^P]GTP was assessed in the presence of: (lane 1 and lane 3) LRRK2 and ARHGEF7 (0 min and 15 min, respectively), (lane 2) LRRK2 and bovine serum albumin (15 min), and (lane 4) ARHGEF7 alone (15 min).

We further investigated the influence of recombinant ARHGEF7 on the GTPase activity of recombinant full-length LRRK2 using a quantitative GTP hydrolysis assay previously described [30]. In our hands, GTP hydrolysis activity of LRRK2 in the presence of bovine serum albumin as negative control is 18% (±2.2) (Figure 7, 15 min lane 2) and can be induced with ARHGEF7 to 36.8% (±4.7) (Figure 7, 0 min lane 1, 15 min lane 3). In comparison, GTP hydrolysis activity of ARHGEF7 alone is 4.9% (±0.2) (Figure 7, 15 min lane 4). The GTP hydrolysis assay points towards an ARHGEF7-mediated, significant twofold induction of LRRK2 GTPase activity. Both the GTP hydrolysis assay and the GTP exchange assay support the notion that ARHGEF7 might be the first identified GEF for LRRK2.

### LRRK2 phosphorylates ARHGEF7 *in vitro*

**Figure 8:**
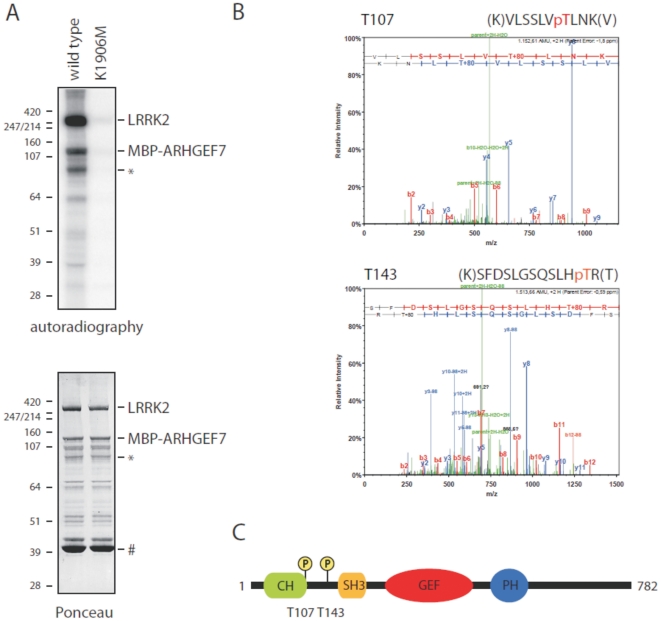
LRRK2 phosphorylates ARHGEF7 at threonine residues within its N-terminus. (A) *In vitro* kinase assay by incubating either WT (lane 1) or kinase-dead (K1906M) LRRK2 (lane 2) with ARHGEF7. Upper panel: Prior to autoradiography samples were blotted onto a PVDF membrane. The upper bands correspond to LRRK2 autophosphorylation, the lower bands to phosphorylation of MBP-tagged ARHGEF7. Lower panel: loading control by ponceau staining of the membrane. Fragments of the ARHGEF7 MBP-fusion protein are indicated by asterisks. A fragment corresponding to the MBP tag only (indicated by #) is not phosphorylated demonstrating that LRRK2 specifically phosphorylates sites within ARHGEF7. (B) Identification of phosphorylated sites within ARHGEF7 by tandem mass spectrometry. MSA-spectra corresponding to tryptic phosphopeptides are shown indicating that ARHGEF7 phosphorylation by LRRK2 occurs at the threonine residues T107 and T143. (C) Graphical representation of the identified phosphorylation sites between the calponin homology domain (CH) and the SH3 domain in the N-terminus of ARHGEF7.

Since LRRK2 interacts with ARHGEF7 we investigated if ARHGEF7 is also a substrate of LRRK2. In order to address this question we performed an *in vitro* kinase assay [9] by incubation of purified wild-type LRRK2 with ARHGEF7, which has been recombinantly expressed as MBP-fusion protein in *E. coli*. The kinase-dead variant K1906M served as negative control to confirm specificity of the observed signals. The Tag-only negative control shows no signal as previously published by Gloeckner et al. 2009 [9]. As shown in Figure 8A, wild-type LRRK2 phosphorylates ARHGEF7. In order to identify the exact location of LRRK2 phosphorylated residues within ARHGEF7 we conducted a mapping approach by mass spectrometry. Two threonine residues, T107 and T143, within the ARHGEF7 N-terminus were identified with high confidence. The spectra of the corresponding tryptic peptides are shown in Figure 8B. The phosphorylation site at T143 was additionally confirmed by a peptide gained from chymotryptic proteolysis (data not shown). The identified LRRK2 phosphorylation sites are located between the calponin homology (CH) and the SH3 domain of ARHGEF7 (Figure 8C).

### N1437S is a potentially new pathogenic mutation within LRRK2

Within a small family with autosomal dominant Parkinson's disease (Figure S5) we identified the new potentially pathogenic mutation N1437S segregating with the disease. The N1437 residue is highly conserved, four amino acids away from the R1441C/G hotspot and was not found in 1000 control chromosomes. The recent finding of the N1437H variant as being pathogenic from Aasly et al. 2010 [33] underlines the central importance of the GTPase domain for the function and the disease pathogenesis of LRRK2 associated disease.

**Figure 9:**
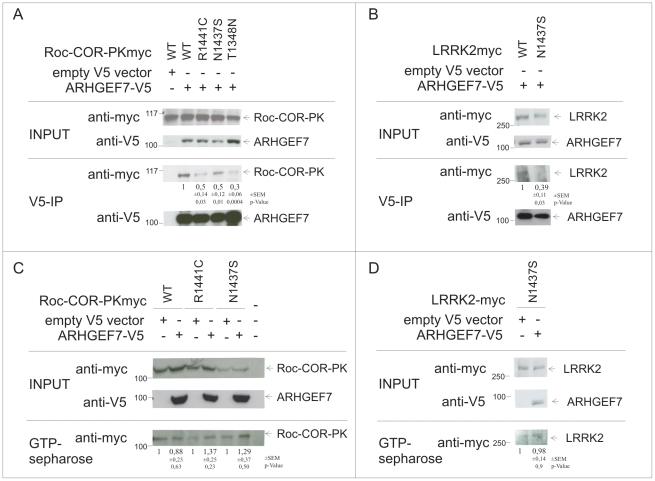
Co-immunoprecipitation of LRRK2 with ARHGEF7 (A/B) and LRRK2-GTP-binding influence through ARHGEF7 (C/D). (A) Myc-tagged Roc-COR-PK Fragment of LRRK2 with indicated GTPase influencing mutations were co-transfected with V5-tagged ARHGEF7 in HEK293 cells and analyzed in V5-Co-immunoprecipitation (V5-IP). 60 µg of protein lysate were used as input control. Immunoblotting was performed using V5 and myc antibody. Empty V5 vector was used as negative control for each condition. Binding of Roc-COR-PK with mutations to ARHGEF7 is indicated in relation to WT Roc-COR-PK with SEM and p-Value. (B) Myc-tagged full-length N1437S-LRRK2 was co-transfected with V5-tagged ARHGEF7 in HEK293 cells and analyzed in V5-Co-immunoprecipitation (V5-IP). 60 µg of protein lysate were used as input control. Immunoblotting was performed using anti V5 and myc antibodies. Binding of N1437S LRRK2 to ARHGEF7 is indicated in relation to WT LRRK2 with SEM and p-Value. (C) GTP-binding efficiency of myc-tagged Roc-COR-PK Fragment (WT, R1441C, N1437S) of LRRK2 on GTP-sepharose was analysed in comparison between co-expression of empty V5 vector or V5-tagged ARHGEF7. Immunoblotting was performed with V5 and myc antibodies. Differences were analysed by pixel densitometry and were indicated. Errors are SEM. *p-value ≤0.05. (D) GTP-binding efficiency of full-length myc-tagged N1437S LRRK2 on GTP-sepharose was analysed in comparison between co-expression of empty V5 vector or V5-tagged ARHGEF7. Immunoblotting was performed with anti V5 and myc antibodies. Differences were analysed by pixel densitometry and were indicated. Errors are SEM. *p-value ≤0.05.

Using a LRRK2 fragment containing the Roc, COR and kinase domains (Roc-COR-PK) we first analyzed the interaction of wild-type and mutant LRRK2 Roc-COR-PK (R1441C, N1437S and T1348N) with wild-type ARHGEF7 in HEK293 cells. As can be seen in Figure 9A there is a decreased interaction of over-expressed mutant LRRK2 with ARHGEF7. The reduced interaction is even more profound using full-length N1437S mutant LRRK2 (Figure 9B) and comparable to the reduced interaction between full-length R1441C-LRRK2 and ARHGEF7.

Over-expression of full-length R1441C-LRRK2 with ARHGEF7 leads to a significant two-fold increase in GTP binding affinity of R1441C-LRRK2 (Figure 5). This can not be observed with the N1437S Roc-COR-PK fragment (Figure 9C) and not with full-length N1437S LRRK2 (Figure 9D) pointing towards different mechanisms exhibited by the two different LRRK2 mutations R1441C and N1437S, respectively.

## Discussion

Even years after the initial description of LRRK2 mutations as a frequent cause of familial Parkinson's disease, only little is known on the normal function of this multidomain protein GTPase and kinase. Since LRRK2-associated disease closely mimics sporadic idiopathic Parkinson's disease with respect to clinical findings, progression, and pathology, there is particular interest in understanding the mechanisms underlying LRRK2 and its pathogenic variants.

After successful cloning and expression of the protein in cell lines, GTPase as well as kinase activity could be demonstrated *in vitro* [7], [8], [12], [20], [34], [35], [36]. Several potential substrates have been described, most convincingly LRRK2 as being its own substrate subjected to autophosphorylation [8], [11], [15]. Recently, phosphorylation sites have been identified in the GTPase domain [14], [15], [17]. This is an important finding and the functional relevance with potential impact on dimerization, GTPase and kinase activity is eagerly awaited.

There is evidence from different studies demonstrating that LRRK2 might influence and regulate neurite outgrowth [25], [26]; however, the underlying mechanisms are yet unknown. Abeliovich and colleagues observed increased neurite outgrowth, a process which is dependent on cytoskeletal dynamics, after viral shRNA knockdown of LRRK2 [26]. Here we propose a mechanism by which LRRK2 might execute its influence on the actin cytoskeleton.

The cytoskeleton plays an important role in maintenance of shape, building protrusions and movement of the cell in general. Central regulators of the cytoskeleton are small GTPases namely, CDC42, Rho and Rac. In order to become activated, GTPases require guanine nucleotide exchange factors (GEFs) that allow switching between GDP and GTP bound states. Of interest, CDC42 is activated by the guanine nucleotide exchange factor ARHGEF7 [37], leading to polymerization of actin and inducing protrusion of the cell membrane. Both genes, CDC42 and ARHGEF7, are significantly up-regulated after acute LRRK2 knockdown by siRNA in SH-SY5Y cells [24]. This might be a possible explanation for the increase in neurite outgrowth after acute knockdown in rat embryos observed by MacLeod and colleagues. To underline the possible link between LRRK2 and both ARHGEF7 and CDC42, we decided to perform co-immunoprecipitation experiments. LRRK2 interacts with ARHGEF7 and CDC42, as can robustly be shown *in vitro* in cell lines and *in vivo* in mouse brain lysates. Evidence for a functional relevance of this interaction is provided by partial co-localization in SH-SY5Y cells.

Several questions became apparent. We first asked if ARHGEF7 might act as a guanine nucleotide exchange factor for the role of LRRK2 as GTPase. To address this question step by step we compared binding of LRRK2 mutants with ARHGEF7 *in vitro*. Over-expression of wild-type and mutant LRRK2 (R1441C, N1437S, G2019S, T1348N and K1906M) in HEK293 cells points towards a reduced binding of R1441C and N1437S mutant LRRK2 with ARHGEF7. The interaction between ARHGEF7 and kinase impaired mutants remains unchanged. This might indicate that the interaction depends on the GDP bound state of LRRK2. The R1441C variant exhibits a reduced GTP hydrolysis activity [20], [36] and should therefore preferably remain in a GTP bound state. ARHGEF7 acts as GEF and is able to convert a GDP bound protein to the GTP bound state, resulting in a preferred binding to GDP bound proteins. In the R1441C LRRK2 condition less GDP bound LRRK2 will be available leading to a reduced binding signal to ARHGEF7. Next, we investigated if co-expression of LRRK2 and ARHGEF7 leads to an increase of the affinity of LRRK2 towards GTP. Only with the R1441C mutant an increased affinity of LRRK2 towards GTP could be demonstrated. This finding additionally points to ARHGEF7 acts as GEF for LRRK2. All of the available GDP bound R1441C LRRK2 proteins will be converted to the GTP bound state, will bind to GTP-sepharose and could not be hydrolysed back in solution as efficiently as in the WT condition. Therefore the enhanced GTP binding of R1441C LRRK2 under influence of ARHGEF7 occurs. Then, we applied a competition assay to analyse the dynamic impact of the ARHGEF7 - LRRK2 interaction. A GEF-dead variant of ARHGEF7 that is still able to bind LRRK2, serves as control. As was expected, co-expression of full-length myc-tagged LRRK2 and empty V5 vector leads to reduced binding of LRRK2 to GTP-sepharose with increasing amounts of soluble GTP. However, co-expression of full-length myc-tagged LRRK2 with wild-type ARHGEF7 leads to reduced LRRK2 binding on GTP-sepharose beads at already much lower concentrations of soluble GTP whereas co-expression of full-length myc-tagged LRRK2 with a GEF-dead variant of ARHGEF7 does not enhance GTP exchange of GTP bound LRRK2. The GTP hydrolysis assay points to a twofold increase of LRRK2 GTPase activity in the presence of ARHGEF7. We therefore conclude that at least *in vitro* ARHGEF7 act as the first identified LRRK2-GEF.

Since LRRK2 interacts with ARHGEF7 and ARHGEF7 is known to be phosphorylated on several sites we also asked if ARHGEF7 might be a substrate of the kinase LRRK2. In order to address this question an *in vitro* kinase assay was performed and the phosphorylation sites were mapped by tandem mass spectrometry. Most interestingly LRRK2 phosphorylates ARHGEF7 at positions previously unknown as being targets for phosphorylation: T107 and T143.

**Figure 10:**
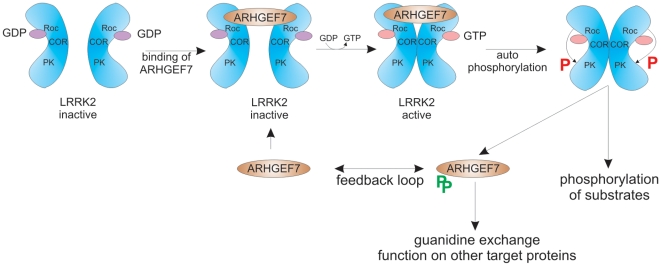
Model of the intermolecular regulation of ARHGEF7 and LRRK2. ARHGEF7 binds as guanine nucleotide exchange factor on dimeric GDP bound LRRK2. Subsequently the GDP-GTP exchange leads to activation of intrinsic GTPase activity of LRRK2, which induce autophosphorylation followed by kinase activation of LRRK2. Kinase active LRRK2 recognizes different substrates among them ARHGEF7.

Although this adds another layer of complexity and awaits further validation *in vivo*, we propose the following model (Figure 10):

ARHGEF7 serves as a guanine nucleotide exchange factor for LRRK2 through mediating the exchange of a GDP to a GTP bound state and by this activating LRRK2. Active LRRK2 is present as autophosphorylated dimer and in this conformation is capable of phosphorylating potential substrates. One substrate is ARHGEF7. ARHGEF7 acts then as a feedback control on LRRK2 activity but also functions on other target proteins like CDC42.

The discovery of pathways in which LRRK2 is involved will hopefully lead to a better understanding of the pathomechanisms of familial and sporadic forms of Parkinson's disease. The identification of crucial proteins within these pathways, positions these proteins as targets for new therapeutical strategies.

## Materials and Methods

### Plasmid Constructs

Full-length human LRRK2 construct was amplified from the pcMV_XL4_Park8 Vector (Origine, Rockville, USA) and cloned in a pcDNA™3.1/myc-His vector (Invitrogen). The G2019S, K1906M, R1441C, N1437S and T1348N mutation were introduced by site-directed mutagenesis (QuikChange™ XL Site-Directed Mutagenesis Kit, Stratagene, La Jolla, USA). Roc-COR-PK (WT, R1441C, N1437S and T1348N) constructs (aa1330-2147) were amplified from the corresponding full-length LRRK2 Vector and introduced in pcDNATM3.1/myc-His vector (Invitrogen) by usage of BamHI and XhoI. HEK293 cDNA (prepared in our lab) was used for amplification and cloning of ARHGEF7 (ENSG00000102606/ENST000000317133) and CDC42 (ENSG00000070831/ENST00000315554) constructs in pCMV-vector thereby introducing a V5-tag at the C-terminus. Generation of the GEF-dead variant (L386R/L387S) of the ARHGEF7 construct [32] was performed by a two step site-directed mutagenesis by using the oligonucleotide *GATAAATACCCTACGCGGCTCAAAGAGCTCGAGA* to introduce the L386R and thereafter the oligonucleotide *GATAAATACCCTACGCGGTCCAAAGAGCTCGAGAGACA* to introduce the L387S mutation. For *in vitro* kinase assays LRRK2 variants were subcloned into the N-SF-TAP vector [38]. For the generation of maltose-binding protein MBP fusion protein, full-length ARHGEF7 was subcloned into the pMAL Vector [16]. For *in vitro* LRRK2 GTPase assays, ARHGEF7 was subcloned into the SF-TAP vector, allowing efficient purification of full length ARHGEF7 from HEK293T cells.

### Antibodies and other reagents

The following antibodies were used in these studies: mouse monoclonal anti-c-Myc (#sc-40, WB = 1∶200, Santa Cruz Biotechnology), anti-V5 rabbit polyclonal antibody (#V8137, WB = 1∶5000, Sigma-Aldrich), anti-LRRK2 rabbit polyclonal antibody (#NB300-267, WB = 1∶1000, IF = 1∶200, Novus Biologicals), rat monoclonal anti-LRRK2 antibody [27], mouse polyclonal ARHGEF7 antibody (#H00008874-A01, WB = 1∶1000, IF = 1∶100 Novus Biologicals), mouse monoclonal CDC42 antibody [M152] (#ab41429, WB = 1∶500, IF = 1∶100, Abcam) and anti-β-Actin mouse monoclonal antibody (Clone AC15, WB = 1∶10,000, IF = 1∶5000, Sigma-Aldrich). Myc-agarose beads (A 7470) and V5-agarose beads (A 7345) for immunoprecipitation studies were purchased from Sigma-Aldrich. Gamma-aminohexyl-GTP-sepharose (Jena Biosciences) was used for GTP binding and GTP competition assays.

### Cell transfection

HEK293 cells (ACC 305) and SH-SY5Y cells (ACC 209) were purchased from DSMZ. HEK293 cells were maintained in D-MEM (+4500 mg/l Glucose, +GlutaMAX™, -Pyruvat, Gibco) and 10% FCS (Gibco) and 1% penicillin/streptomycin (Gibco). SH-SY5Y cells were grown under the same conditions accepts of 15% FCS. HEK293 cells were transiently transfected by using Lipofectamine 2000 (Invitrogen) according to the manufacturer's instructions. RNA Interference of LRRK2 in SH-SY5Y cells was performed as previously described [24].

### Immunoprecipitation and immunoblotting

Protein lysis for immunoprecipitation studies were performed 48 h after transiently transfection with 150 µl NP40-Lysisbuffer (PBS +0,5% NP40 +0,5% deoxycholate) with freshly added complete protease inhibitor cocktail (Roche Molecular Biochemicals) and phosphatase inhibitor cocktail I and II (Sigma Aldrich). Therefore cells were incubated 30 min on ice while vortexing each 10 min following by 15 min centrifugation at 4°C at 13.000 rpm. Protein concentration of the supernatant was analyzed by the Bradford method. 1000 µg of protein lysate was used for immunoprecipitation with either myc-agarose beads or V5-agarose beads. After rotating the beads over night at 4°C, Pellet was washed once with NP40-Lysisbuffer and three times with PBS. Proteins bound to the agarose beads were eluted with 20 µl 2xLaemmli buffer and 10 min incubation at 70°C. The whole lysate was loaded on SDS-PAGE. As input control 60 µg of the protein lysate was used.

### Immunoprecipitation from mouse brain

Protein lysis of whole brain of C57Bl6 WT mice was performed using 1.5 ml PBS with 1% TritonX100 and freshly added complete protease inhibitor cocktail (Roche Molecular Biochemicals) and phosphatase inhibitor cocktail I and II (Sigma Aldrich) with a dounce -homogenisator. After 45 min incubation with rotation at 4°C the lysate was centrifuged for 15 min at full speed. The concentration of the supernatant was analyzed by the Bradford method. For coupling of the endogenous proteins to the specific LRRK2 antibody (rat monoclonal anti-LRRK2 1E11, IP = 1∶200, generated by E. Kremmer, Helmholtz Zentrum München, Munich, Germany) 3000 µg of protein lysate was incubated in a volume of 200 µl for 3 h with rotating at 4°C. As negative control normal rat IgG (PeproTech, 1∶200) was coupled to the beads. Thereafter 40 µl of ProteinG-agarose beads (#P4691 Sigma Aldrich) were added for another 2 h. The beads were washed once with the lyses buffer and twice with PBS before adding 25 µl 2xLaemmli buffer to the beads. The samples were incubated 10 min at 70°C and loaded on SDS-PAGE. For detection the following antibodies were used: mouse polyclonal ARHGEF7 antibody (#H00008874-A01, WB = 1∶1000, Novus Biologicals), mouse monoclonal CDC42 antibody [M152] (#ab41429, WB = 1∶500, Abcam), mouse anti-SOS1 (#610095, WB = 1∶250, IP = 1∶25, BD Transduction Laboratories), Anti-α-Tubulin Mouse mAb (#CP06, WB = 1∶50000, Calbiochem) and rat monoclonal anti-LRRK2 (1E11, WB = 1∶1000 [27]. We obtained ethics approval for protein lysis of whole brain of C57Bl6 WT mice by the “Regierungspräsidium Tübingen”.

### GTP binding assay

GTP binding assay was performed as described by West et al. except of using 150 µl lysis buffer G per 6well of 48h transiently transfected HEK293 cells [12]. 800 µg of each lysate was incubated for 3 h on 20 µl GTP-sepharose while rotating at 4°C. 2 mM GDP was added for another 1 h rotating. GTP-sepharose was washed once with lysis buffer G and two times with PBS. GTP-bound proteins were eluted with 20 µl 2xLämmli buffer, incubated for 10 min at 70°C and completely loaded on SDS-PAGE. As input control 60 µg of the protein lysate was used. The amount of GTP-bound LRRK2 was normalized to input levels.

### GTP competition assay

GTP competition analysis was done as described for GTP binding assay. 4 wells of transfected HEK293 cells were lysed and pooled. For each competition condition (0, 0.5, 1, 2, 4 mM GTP) 300 µg of lysate was incubated for 1 h on 20 µl GTP-sepharose beads. Afterwards the specified amount of GTP was added and the incubation was performed for one additional hour.

### GTP hydrolysis assay

GTP hydrolysis assays based on thin-layer chromatography were performed as described in detail elsewhere [30]. Briefly, recombinant full-length LRRK2 (50 nM) and/or ARHGEF7 (75 nM) were pre-incubated with 500 nM [α-^33^P]GTP (3000Ci/mmol) in 20 M Tris, pH 8.0, 1 mM EDTA, 2 mM dithiothreitol, for 5 min at room temperature. The reaction was started by adding MgCl_2_ (final concentration 5 mM) and incubating at 37°C. At 0 min and 15 min the reaction was stopped by mixing with a solution containing 0.2% (v/v) sodiumdodecylsulfate, 2 mM EDTA, 2 mM dithiothreitol, 0.5 mM GTP, 0.5 mM GDP and heating to 65°C. 2 µl aliquots were spotted onto thin-layer plates (Merck, Darmstadt, Germany) and separated in 1 M KH_2_PO_4_ buffer, pH 3.5. The plates were exposed to phosphoscreens overnight. The screens were imaged on a Typhoon 9400 laser scanner (GE Healthcare, Freiburg, Germany) and the radiolabeled spots were quantified using Quantity One software (Bio-Rad, Munich, Germany).

### Statistical analyses

For interaction analyses, GTP binding and competition assays the intensities of protein bands were analyzed with the AlphaEaseFC™ software (Alpha Innotech). After background correction and normalization with an unspecific band the pixel intensity of each protein was related to the corresponding control protein band. Statistics were done using the student's t-test.

### Immunofluorescence

SH-SY5Y cells were differentiated 6 days with 10 µM retinoic acid [39] and subsequently fixed with 4% paraformaldehyde in PBS. After 5 min incubation with icecold methanol the cells were washed twice with PBS and blocked with 10% normal donkey serum for 30 min. Incubation with primary antibodies was performed over night at 4°C. After three-times washing with PBS, 1 h incubation with the labeled Cy2 and Cy3 secondary antibody at 37°C the cells were mounted with DABCO/Mowiol. The confocal images were taken with the LSM 510 (Laser Scanning Microscope, Carl Zeiss MicroImaging GmbH) by using a pinhole Ø 1,00 Airy Units (corresponding an optical slice <0,8 µm) without using Z-stacks.

### Kinase Assay

For the *in vitro* kinase assay, N-terminal SF-TAP tagged wild-type LRRK2 and kinase-dead LRRK2 (K1906M) were purified via tandem affinity purification as described in [40]. As substrate MBP-tagged ARHGEF7 purified from *E. coli* has been used. Purification of the MBP fusion protein was performed as described earlier [9]. The *in vitro* kinase assay using ^32^P labelled ATP was performed as described recently [9]. Briefly, SF-TAP tagged LRRK2 variants bound to anti-FLAG agarose (Sigma) were incubated with 2 µg of MBP-ARHGEF7 in 30 µl assay buffer (25 mM Tris-HCl pH 7.5, 5 mM beta-glycerophosphate, 2 mM DTT, 0.1 mM Na_3_VO_4_, 10 mM MgCl_2_, Cell Signalling) supplemented with 50 µM MgATP (Cell Signalling) and 3 µCi Redivue γ^32^P-ATP (GE-Healthcare). The reaction mix was incubated for 2 h at 30°C. The reaction was stopped by addition of 10 µl 5x Laemmli buffer and samples were incubated for two minutes at 96°C prior to SDS gel-electrophoresis and immunoblotting. Autoradiograms were obtained by exposure of phospho-imager plates to the membranes which were then quantified by a Typhoon Trio Reader (GE-Healthcare).

### Mapping of phosphorylation sites by mass spectrometry

For the identification of the ARHGEF7 phosphorylation sites, the kinase assay was repeated with 100 µM non-labelled ATP. Sample preparation for mass spectrometry was performed as described earlier [40]. Briefly, proteins were precipitated by chloroform/methanol. The protein precipitates were dissolved in 50 mM ammonium bicarbonate, containing 0.2% RapiGest [41], reduced with DTT and alkylated with iodoacetamide and proteolysis was performed by incubation with 2 µg of trypsin (Promega) o/n at 37°C. After proteolysis the RapiGest surfactant was hydrolysed by adding HCl and incubation for 30 min at RT. Samples were centrifuged for 10 min (13.000× g, RT) and the supernatants were transferred to new vials. Sample volumes were reduced to approx. 10 µl in a speed vac. Phosphopeptide enrichment was performed by TiO_2_ using protocols published recently [42]. Mass spectrometric analysis of the samples was performed on an LTQ OrbitrapXL mass spectrometer (Thermo-Fisher) coupled to an Ultimate 3000 Nano-HPLC (Dionex). In addition to MS/MS, multistage activation [43] was used for PTM analysis. Database search was performed against the Uniref 100 database (date: 2009-06-08) using the Mascot search engine (version: 2.2.06, Matrix Science) with following parameters: trypsin as enzyme, 10 ppm as mass range for parent ions and 1 Da for fragment ions; carbamidomethyl as fixed modification and methionine oxidation and serine/threonine as well as tyrosine phosphorylation as variable modifications.

## Supporting Information

Figure S1 Specificity of the LRRK2 antibody. Specificity of the human-specific anti-LRRK2 antibody, used for immunofluorescence analysis, is shown by RNAi mediated knockdown of LRRK2 (siLRRK2-1) in SH-SY5Y cells in comparison to control siRNA transfected cells.
(0.18 MB TIF)

Figure S2 Specificity control for used immunoprecipitation approach: endogenously expressed LRRK2 shows interaction with Tubulin but not with the GEF SOS1. Endogenous LRRK2 coupled on ProteinG agarose beads is not able to pull down the GEF SOS1 from mouse brain lysate, and the coupling of endogenous SOS1 shows no interaction to endogenous LRRK2 (A). The previously known interaction partner Tubulin Alpha (TUBA) could be confirmed as endogenous interacting protein of LRRK2 in mouse brain lysate (B).
(0.60 MB TIF)

Figure S3 Quantification of the LRRK2 Interaction (WT and mutations) with ARHGEF7. Pixel densities of three independent experiments of interaction analyses between LRRK2 with mutations and ARHGEF7 (Figure 4) were calculated in relation to interaction between LRRK2 (45) and ARHGEF7.
(0.16 MB TIF)

Figure S4 Quantification of LRRK2 binding to GTP influenced by ARHGEF7. Pixel densities of three independent experiments of GTP-binding of mutated or WT LRRK2 in presence of ARHGEF7 (Figure 5) were calculated in relation to the GTP-binding of mutated or WT LRRK2 in presence of empty V5-vector.
(0.17 MB TIF)

Figure S5 Pedigree of a family with Parkinson's disease. The new potentially pathogenic mutation N1437S is segregating with disease.
(0.10 MB TIF)
