# Supplementary figures and images for: ARHGEF7 (BETA-PIX) Acts as Guanine Nucleotide Exchange Factor for Leucine-Rich Repeat Kinase 2
Source: PLoS One. 2010 Oct 29;5(10):e13762. doi: 10.1371/journal.pone.0013762 (PMC2966438; doi:10.1371/journal.pone.0013762)

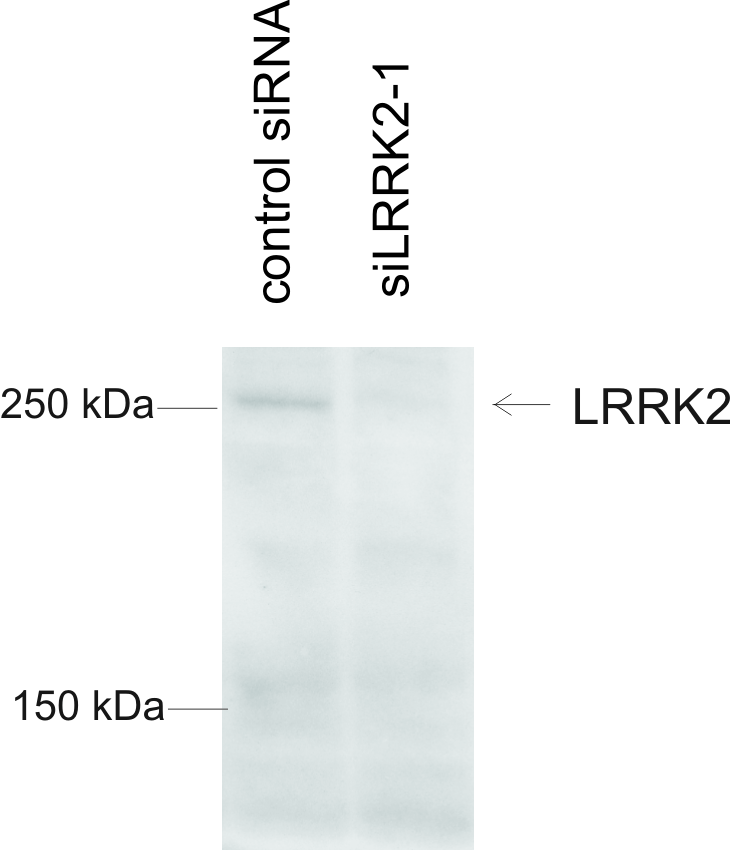

Supplement: Figure S1 — Specificity of the LRRK2 antibody. Specificity of the human-specific anti-LRRK2 antibody, used for immunofluorescence analysis, is shown by RNAi mediated knockdown of LRRK2 (siLRRK2-1) in SH-SY5Y cells in comparison to control siRNA transfected cells. (0.18 MB TIF) [file pone.0013762.s001.tif]

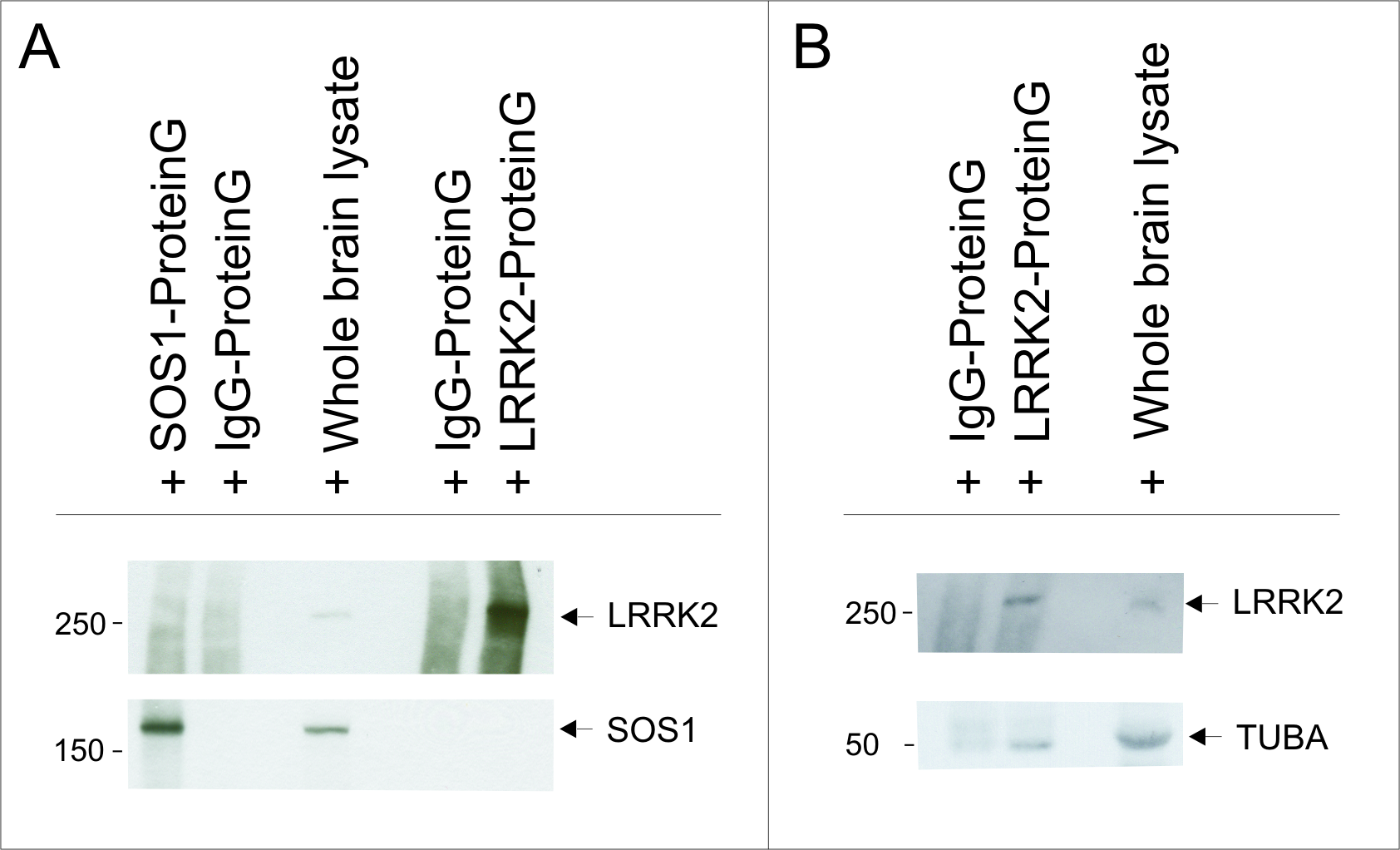

Supplement: Figure S2 — Specificity control for used immunoprecipitation approach: endogenously expressed LRRK2 shows interaction with Tubulin but not with the GEF SOS1. Endogenous LRRK2 coupled on ProteinG agarose beads is not able to pull down the GEF SOS1 from mouse brain lysate, and the coupling of endogenous SOS1 shows no interaction to endogenous LRRK2 (A). The previously known interaction partner Tubulin Alpha (TUBA) could be confirmed as endogenous interacting protein of LRRK2 in mouse brain lysate (B). (0.60 MB TIF) [file pone.0013762.s002.tif]

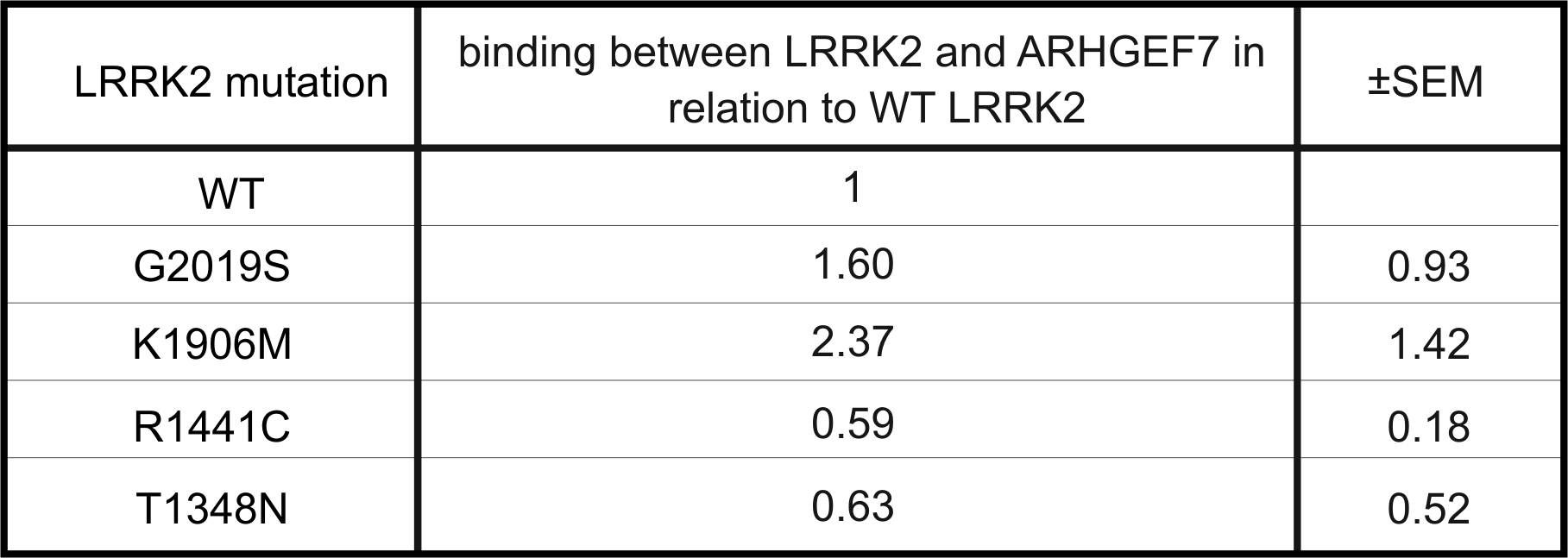

Supplement: Figure S3 — Quantification of the LRRK2 Interaction (WT and mutations) with ARHGEF7. Pixel densities of three independent experiments of interaction analyses between LRRK2 with mutations and ARHGEF7 (Figure 4) were calculated in relation to interaction between LRRK2 (45) and ARHGEF7. (0.16 MB TIF) [file pone.0013762.s003.tif]

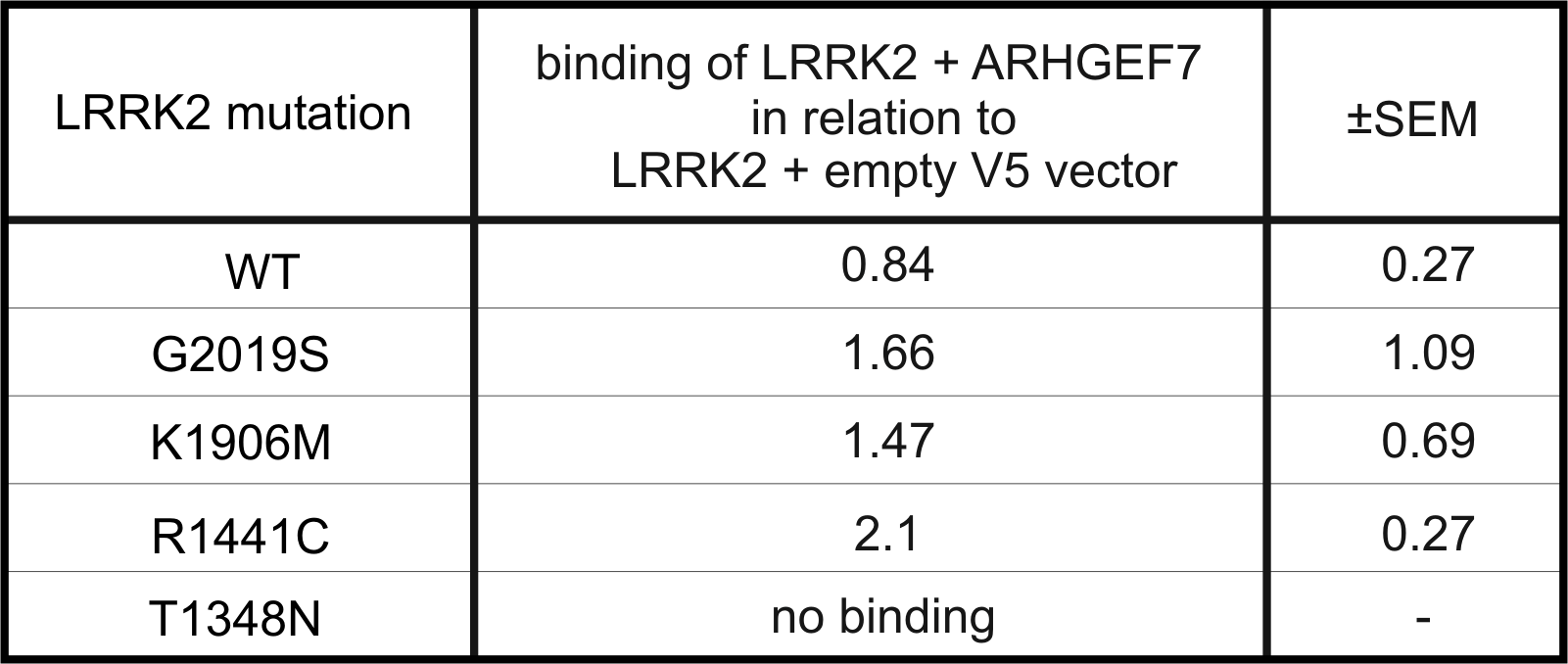

Supplement: Figure S4 — Quantification of LRRK2 binding to GTP influenced by ARHGEF7. Pixel densities of three independent experiments of GTP-binding of mutated or WT LRRK2 in presence of ARHGEF7 (Figure 5) were calculated in relation to the GTP-binding of mutated or WT LRRK2 in presence of empty V5-vector. (0.17 MB TIF) [file pone.0013762.s004.tif]

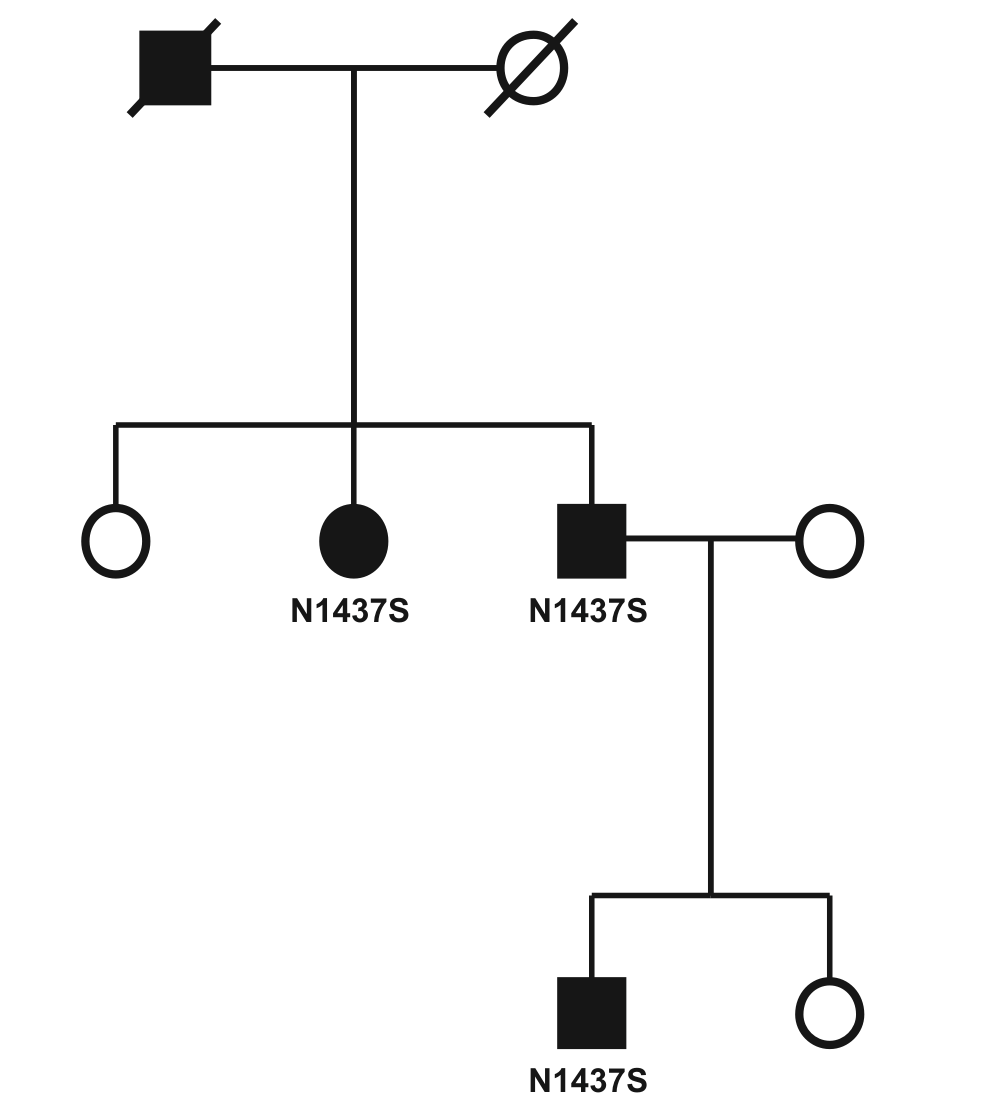

Supplement: Figure S5 — Pedigree of a family with Parkinson's disease. The new potentially pathogenic mutation N1437S is segregating with disease. (0.10 MB TIF) [file pone.0013762.s005.tif]
